# Phylogenetic reconciliation: making the most of genomes to understand microbial ecology and evolution

**DOI:** 10.1093/ismejo/wrae129

**Published:** 2024-07-13

**Authors:** Tom A Williams, Adrian A Davin, Lénárd L Szánthó, Alexandros Stamatakis, Noah A Wahl, Ben J Woodcroft, Rochelle M Soo, Laura Eme, Paul O Sheridan, Cecile Gubry-Rangin, Anja Spang, Philip Hugenholtz, Gergely J Szöllősi

**Affiliations:** School of Biological Sciences, University of Bristol, Bristol BS81TQ, United Kingdom; Department of Biological Sciences, Graduate School of Science, The University of Tokyo, 113-0033 Tokyo, Japan; MTA-ELTE “Lendület” Evolutionary Genomics Research Group, Eötvös University, 1117 Budapest, Hungary; Model-Based Evolutionary Genomics Unit, Okinawa Institute of Science and Technology Graduate University, 904-0495 Okinawa, Japan; Biodiversity Computing Group, Institute of Computer Science, Foundation for Research and Technology Hellas, 70013 Heraklion, Greece; Computational Molecular Evolution Group, Heidelberg Institute for Theoretical Studies, 69118 Heidelberg, Germany; Institute of Theoretical Informatics, Karlsruhe Institute of Technology, 76131 Karlsruhe, Germany; Biodiversity Computing Group, Institute of Computer Science, Foundation for Research and Technology Hellas, 70013 Heraklion, Greece; Centre for Microbiome Research, School of Biomedical Sciences, Queensland University of Technology (QUT), Translational Research Institute, Woolloongabba, QLD 4102, Australia; Australian Centre for Ecogenomics, School of Chemistry and Molecular Biosciences, The University of Queensland, Brisbane, QLD 4072, Australia; Unité d’Ecologie, Systématique et Evolution, Université Paris-Saclay, 91190 Gif-sur-Yvette, France; School of Biological and Chemical Sciences, University of Galway, Galway H91 TK33, Ireland; School of Biological Sciences, University of Aberdeen, Aberdeen AB24 3FX, United Kingdom; Department of Marine Microbiology and Biogeochemistry, NIOZ, Royal Netherlands Institute for Sea Research, PO Box 59, 1790 AB Den Burg, The Netherlands; Department of Evolutionary & Population Biology, Institute for Biodiversity and Ecosystem Dynamics (IBED), University of Amsterdam, Amsterdam, The Netherlands; Australian Centre for Ecogenomics, School of Chemistry and Molecular Biosciences, The University of Queensland, Brisbane, QLD 4072, Australia; MTA-ELTE “Lendület” Evolutionary Genomics Research Group, Eötvös University, 1117 Budapest, Hungary; Model-Based Evolutionary Genomics Unit, Okinawa Institute of Science and Technology Graduate University, 904-0495 Okinawa, Japan; Institute of Evolution, HUN REN Centre for Ecological Research, 1121 Budapest, Hungary

**Keywords:** phylogenetics, gene tree–species tree reconciliation, microbial evolution, horizontal gene transfer

## Abstract

In recent years, phylogenetic reconciliation has emerged as a promising approach for studying microbial ecology and evolution. The core idea is to model how gene trees evolve along a species tree and to explain differences between them via evolutionary events including gene duplications, transfers, and losses. Here, we describe how phylogenetic reconciliation provides a natural framework for studying genome evolution and highlight recent applications including ancestral gene content inference, the rooting of species trees, and the insights into metabolic evolution and ecological transitions they yield. Reconciliation analyses have elucidated the evolution of diverse microbial lineages, from Chlamydiae to Asgard archaea, shedding light on ecological adaptation, host–microbe interactions, and symbiotic relationships. However, there are many opportunities for broader application of the approach in microbiology. Continuing improvements to make reconciliation models more realistic and scalable, and integration of ecological metadata such as habitat, pH, temperature, and oxygen use offer enormous potential for understanding the rich tapestry of microbial life.

## Introduction

Phylogenetic trees are important in microbial ecology, because to understand an ecosystem you need to understand its evolutionary history across multiple levels, ranging from genes to species to entire communities. For example, horizontal transfer of key genes between members of a community can have a large impact on organismal and community function as a whole [1]. The identification of such genes is often achieved via manual comparisons of species and gene trees to identify putative transfers of interest. Recently, new gene tree–species tree reconciliation methods including RANGER-DTL 2 [2], TALE [3], and AleRax [4] have emerged that take this approach to the next level, allowing thousands of genes to be compared with species trees.

These phylogenetic reconciliation methods have many applications in the study of microbial ecology and evolution, and indeed in biology more broadly, beyond the detection of horizontal gene transfer (HGT). For example, they can be used to infer more accurate species and gene trees and to root them. Furthermore, these inferences can be used to reconstruct ancestral gene content or to evaluate the evidence for coevolution between host and parasite or symbiont lineages. Reconciliation analyses have also been used to improve the reconstruction of ancestral protein sequences for use in evolutionary biochemistry and synthetic biology applications [5], and for studying whole-genome duplication in land plants [6].

As with any kind of data analysis, the accuracy of phylogenetic reconciliation depends on the algorithms used and the assumptions they make about the processes of evolution [7]. There has been substantial progress in method development in recent years [8], including the development of methods that can accommodate uncertainty in the evolutionary history of single genes [9–11], incomplete lineage sorting [12], and that allow the inferred rates of gene duplication, transfer, and loss (DTL) to vary across the species tree [4], resulting in more sophisticated software packages for performing these analyses. Here, we first review how reconciliation methods work and argue that they provide a natural framework for modelling microbial genome evolution. We then review a body of recent work that illustrates how reconciliation methods can provide insight into microbial ecology and evolution.

## Modelling microbial evolution

The application of phylogenetics to the study of microbial ecology and evolution has proven powerful but does not come without limitations. With the advent of high-throughput sequencing, single-gene alignments often contain more taxa than sites (aligned amino acid or nucleotide positions) and may contain too little information with which to confidently resolve sequence relationships. Even well-supported gene trees are statistical estimates of evolutionary history and are not guaranteed to be correct. In particular, trees depend on the model of sequence evolution used to infer them, which describes the rates of different amino acid or nucleotide substitutions over time (substitution model). Numerous models are available, and model choice is important because poorly fitting models can result in the inference of an incorrect tree with high statistical support [7]. A specific issue for microbial evolution is that existing models, many of which have been in use for decades, were inferred from small datasets predominantly comprising closely related animals and plants. Therefore, these models may not be representative of prokaryotic evolution [13]. Recent work has aimed to develop more appropriate substitution models for prokaryotes and microbes more broadly, with packages such as MAMMaL, EDCluster, and QMaker now available for estimating new models [13–15].

The importance of the substitution model in phylogenetics is exemplified by long-running debates in the literature about the deep structure of the tree of life, in which analyses of the same data using different models resulted in support for either two [16] or three [17] primary domains of life [18]. Best practice in phylogenetics is therefore to evaluate the fit of a range of substitution models to a given dataset and perform inference using the best-fitting model, as judged by statistical tests such as the Bayesian information criterion or the Akaike information criterion. However, better-fitting models are generally more complex and less computationally tractable, such that difficult decisions have to be made about the trade-off between dataset size and model adequacy—the degree to which a model captures the evolutionary process that gave rise to the sequence data. The scalability of phylogenetic methods, both simple and complex, is an increasing challenge in microbial evolution research that aims to integrate the wealth of new genome data being generated by cultivation-free approaches.

HGT, an important driver of microbial evolution [19], presents another modelling challenge. On generation-to-generation timescales, inheritance in prokaryotes is usually vertical, with transmission of the genome from mother to daughter cells [20]. This genetic process gives rise to the species tree describing the relationships among lineages. Among closely related genomes, HGT and homologous recombination can sometimes act to homogenize the gene pool and reinforce species boundaries [21]. However, HGT over longer genetic distances induces differences between gene trees, and between the gene trees and the species tree. The cumulative effect of gene transfer is that very few, if any, prokaryotic genes share the same history as the lineages they reside in [22], with the possible exception of very young (i.e. recently evolved) genes that have not yet experienced transfer. When viewed on a long evolutionary timescale, lineages of prokaryotes (and, perhaps, microbial eukaryotes) might be regarded as “ships of Theseus”, continuously remodelled by gene transfer despite clear continuity of inheritance from one generation to the next [23].

In addition to HGT, gene duplication and loss are fundamental processes that shape microbial genomes and need to be taken into account in any model. Duplicated genes are particularly common in eukaryotes [24] but are important for the emergence of novelty in all lifeforms, with duplicated genes experiencing less selective constraints that can enable the evolution of new functions [25]. Ancient gene duplications underpinned the evolution of core molecular complexes of prokaryotic cells, including the membrane-bound ATP synthase [26, 27] and the signal recognition particle/receptor system for targeting proteins to the cell membrane [28], but gene duplication also plays an important role in prokaryotes on more recent timescales. For example, exposure to antibiotics can promote the fixation of duplicate resistance genes [29] as a means of increasing gene dosage and therefore expression level [30], and gene duplication followed by functional divergence has been shown to drive the evolution of biosynthetic gene clusters that produce novel secondary metabolites in *Streptomyces* [31].

Gene loss is frequent in both prokaryotes and eukaryotes and underpins the evolution of symbiotic and parasitic lineages, including the nutritional endosymbionts of aphids [32], DPANN Archaea(“DPANN” Archaea were originally defined as a superphylum containing Diapherotrites, Parvarchaeota, Aenigmaarchaeota, Nanoarchaeota, and Nanohaloarchaeota [33], but now also contain other small-genome lineages such as Woesearchaeota and Pacearchaeota [34].) [33, 34], Patescibacteria/CPR [35, 36], and parasitic fungi such as Microsporidia [37]. Although the lost genes often encode metabolic functions that are no longer required following the shift to a host-associated lifestyle, it has also been suggested that periods of gene loss may facilitate subsequent adaptive evolution via the disruption of preexisting gene interaction networks [38].

**Figure 1:**
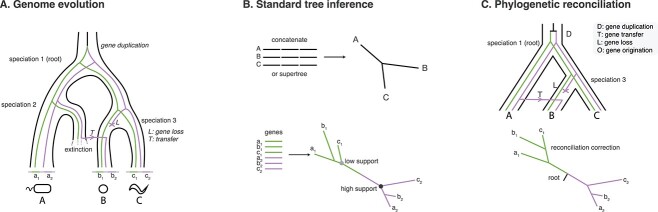
Modelling microbial evolution using phylogenetic reconciliation. (A) A conceptual model of microbial genome evolution, showing an overarching species (or lineage) tree with three speciations and one extinction event. Genes evolve along this tree, occasionally jumping between lineages by HGT (arrow), being duplicated (triangle), or being lost (cross). A, B, and C are different species; a_1_ and a_2_ are homologous genes that are part of the broader gene family being analysed, and which are both found in species A. (B) Standard phylogenetic inference reconstructs the tree from the sequence alignment using a stochastic substitution model. Single gene alignments are often short and contain limited information, so that the inferred gene tree can contain weakly supported branches. To estimate the species tree, information can be aggregated from multiple genes using concatenation or supertree approaches. However, these approaches estimate the topology but in most cases not the root of the species tree, unless an outgroup is included [117] or a nonreversible model is used [118, 119]. (C) To model microbial evolution, we need to capture not only the evolution of gene sequences along gene trees (via a substitution model) but also the evolution of gene trees along the species tree (via a reconciliation model that describes rates of DTL). The result is a kind of “species tree aware” phylogenetics, in which information from many gene families is used to infer the species tree, and in turn the histories of the individual gene families are contextualized as a series of gene duplication, loss, and transfer events. Phylogenetic reconciliation can combine this information by jointly optimising the phylogenetic (substitution) and reconciliation likelihoods. In the example gene tree in panel B, there is strong support for a sister relationship between the a_2_ and b_2_ genes (in the red clade), indicating a transfer event from Branch A to B on the species tree (Panel C); however, the sister relationship predicted for the a_1_ and b_1_ genes (in the blue clade) is weakly supported and likely incorrect, given the B + C relationship in the species tree. By incorporating information from the species tree, reconciled gene trees can show improved accuracy over the gene tree estimated from the sequence alignment alone [56].

A complete picture of microbial genome evolution therefore requires consideration of HGT, gene duplication, and loss. These processes can be captured via a conceptual model of microbial evolution along the lines of that depicted in Fig. 1A, in which genes evolve vertically along a species tree, sometimes experiencing gene duplications, losses, and transfers into other contemporary lineages. The questions we might want to ask of this model include what is the overarching species tree? What are the relative contributions of vertical transmission and horizontal transfer to genome evolution, and do these vary over the tree? Where in evolutionary history did gene duplications, transfers, and losses occur? Which gene families were present at each internal node on the tree, and consequently which ancestral metabolic capabilities and environmental adaptations can be inferred at each time point?

The first of these questions—the topology of the species tree—can be addressed using concatenation or supertree approaches, whereby phylogenetic information is combined from multiple genes predicted to have been inherited vertically from a common ancestor (Fig. 1B). In the concatenation approach, initial phylogenetic analyses are used to identify a set of genes that, within the limits of statistical resolution, appear to have congruent evolutionary histories. Multiple sequence alignments for each of these genes are then stitched together and analysed as a single alignment, providing a simple way to pool phylogenetic information. User-friendly tools for inferring trees from sequence alignments are now available, with some of the most popular packages including RAxML-NG [39], IQ-TREE [40], and PhyloBayes [41]. By contrast, supertree or “species tree” methods take a two-step approach: single gene trees are inferred separately for each gene (using a program such as RAxML-NG or IQ-TREE) and then their phylogenetic signal is combined using one of a variety of heuristic algorithms [42–44], with ASTRAL and related tools among the most popular and performant methods [45]. An advantage of concatenation methods is that longer alignments allow the use of better-fitting but more complex substitution models, which have repeatedly been shown to be important for accurate inference of phylogenetic trees in deep time [46]. Supertree methods can be faster and are more robust to incomplete lineage sorting and HGT among the input gene trees than is concatenation [47]. It is encouraging that the results of concatenation and supertree analyses are often concordant and, in recent years, have been converging on the topology of the tree of life. For example, concatenation of core genes and supertree analysis of broadly shared gene families both recovered a “two domains” tree of life [48], and taxonomic schemes inferred from large-scale prokaryotic trees inferred using concatenation and supertree methods were 98.2% identical [49].

Beyond species tree estimation, answering the other questions about microbial genome evolution posed above requires comparison of gene and species trees. Manual comparison of individual gene trees with the species tree can identify putative gene transfers, but the approach lacks power because any given gene tree can be explained by many different combinations of DTL events. The problem is compounded by phylogenetic uncertainty, further increasing the number of possible scenarios, and by phylogenetic error. Phylogenetic reconciliation systematically addresses these issues by modelling the evolution of gene families in the shared context of the overarching species tree, capturing both sequence evolution and higher-level processes of DTL.

Figure 1C illustrates how the conceptual model of microbial evolution outlined above and in Fig. 1A can be operationalized using phylogenetic reconciliation methods. Reconciliations are mechanistically explicit scenarios that describe how a gene family has evolved on the species tree, starting with an origination event at a specific ancestral node, and followed by a series of events, each of which is mapped to a specific branch of the species tree. The reconciliation scenario ends with the members of the gene family observed in modern genomes arriving at the tips of the species tree. The set of possible events include vertical transmissions from ancestor to descendant node, gene duplications, transfers (from a donor to a recipient branch), and losses. For a given species tree and gene tree, many distinct reconciliation scenarios are possible, so we need criteria for choosing between them. One approach is to use the principle of parsimony: if we can assign relative costs to the different possible event types in advance (e.g. DTLs), we can find the reconciliation(s) that have the lowest summed cost [2, 50–53]. Alternatively, probabilistic model-based approaches can be used to estimate the rates of each type of event from the data using maximum likelihood or Bayesian methods [4, 9, 10, 54]. These are more computationally intensive but have the advantage that rates do not need to be set *a priori*. What we know of microbial ecology and evolution suggests that the relative rates of events vary across the tree of life. For example, duplications are more frequent in eukaryotes, transfers in prokaryotes, and losses in host-associated lineages [55]. As such, model-based approaches that estimate the DTL rates directly from the data have clear advantages for studying microbial evolution [56]. For example, reconciled gene trees inferred using the probabilistic reconciliation method ALE (amalgamated likelihood estimation [10]) were more accurate than those inferred with a range of parsimony methods [56, 57]. Thus, while analyses with a diversity of reconciliation methods have provided insights into microbial ecology and evolution (see practical applications discussed in the next section and Table 1), our recommendation is that probabilistic model-based methods should be used where possible (see Box 1).

**Table 1:** A selection of available reconciliation software that can model DTL; for a more comprehensive overview of available packages, see [8].

**Package**	**Inference framework**	**Input**	**Remark**	**Repository**	**Reference**
RANGER-DTL2	Parsimony	Single gene tree for each multiple sequence alignment (MSA)	Widely used parsimony reconciliation tool	https://compbio.engr.uconn.edu/software/RANGER-DTL/	[2]
TreeFix DTL	Parsimony	Single gene tree for each MSA	Widely used parsimony reconciliation tool	https://compbio.mit.edu/treefix-dtl/index.html#download	[11]
ecceTERA	Parsimony	Sample of gene trees for each MSA	Reconciliation of gene trees in a parsimony framework	https://mbb.univ-montp2.fr/MBB/subsection/softExec.php?soft=eccetera	[52]
ALE	Probabilistic (maximum likelihood, Bayesian)	Distribution of gene trees for each MSA	Calculate species tree likelihood and sample reconciled gene trees in a probabilistic framework	https://github.com/ssolo/ALE	[10]
TALE	Probabilistic (Bayesian)	Host species tree, single or distribution of symbiont and gene trees	Implements a three-level hierarchical reconciliation approach: genes within symbionts within hosts	https://github.com/hmenet/TALE	[3]
GeneRax	Probabilistic (maximum likelihood)	MSA	Directly estimates ML reconciled gene tree, good for very similar sequences	https://github.com/BenoitMorel/GeneRax	[59]
AleRax	Probabilistic (maximum likelihood)	Distribution of gene trees for each MSA	Calculate species tree likelihood and sample reconciled gene trees in a probabilistic framework	https://github.com/BenoitMorel/AleRax	[4]

Box 1 Best practices in phylogenetic reconciliation.

The most common phylogenetic reconciliation task is mapping the evolutionary histories of a collection of gene families onto a rooted species tree. We describe one workflow using AleRax, a fast and accurate state-of-the-art tool recently developed by some of us, but we encourage interested users to explore the range of tools available. Each of the steps below is itself a large topic, and we refer the interested reader to further literature throughout. The software repository is usually the best resource for information on how to run tools. For example, see https://github.com/BenoitMorel/AleRax/wiki for AleRax or https://compbio.engr.uconn.edu/software/RANGER-DTL/ for RANGER-DTL2.

We will first infer the gene trees, and infer and root the species tree. We recommend using the most accurate methods for each of these steps, to the extent that time and computational resources allow. However, consider using faster, less accurate alignment and tree inference options or trading off dataset size when doing initial analyses, to save time and carbon emissions.

Gene tree inference.
  Work with the nucleotide or protein sequences from a set of genomes of interest and label each with the genome of origin and the unique gene ID. For example, “ECOLI_XBV38467.” By default, AleRax will interpret what comes before the underscore as the species name, and what comes after as the gene ID, which will aid with mapping later.
  Cluster the sequences into gene families, either using established sets of homologous clusters (such as through COG or KEGG annotation of your set of genomes using eggNOG-mapper [123]), or a *de novo* gene clustering tool (such as mcl [124] or Broccoli [125]) Gene families must include all homologous genes, i.e. orthologues, paralogues, and xenologues.
  Align the gene families using e.g. muscle 5 [126].
  Infer trees for each gene family using software such as IQ-TREE2 [40], RAxML-NG [39], PhyloBayes [41], or MrBayes [127]. If using a maximum likelihood program, tell it to write the bootstrap trees to an output file (e.g. “-wbtl” in IQ-TREE2), because it is these—not the maximum likelihood tree—that capture the uncertainty about evolutionary relationships within the gene family. If using a Bayesian tool, use a sample of trees from the posterior distribution to represent this uncertainty. We recommend using a Bayesian tool if dataset size allows, because the conditional clade probabilities used in AleRax benefit from an accurate sample of the posterior distribution, which the maximum likelihood bootstrap can only approximate.
Species tree inference [47, 128].
  Use a best-practice approach to species tree inference—either using a concatenate of universal markers or supertree methods (see main text). There are several established tools for obtaining marker genes, including BUSCO [129], GTDB-Tk [130], or OrthoFinder [131].
  Root the species tree based on prior evidence or using an appropriate rooting method; e.g. in a study of opisthokonts, you might root the tree between Holozoa and Holomycota. If you are unsure of the root position, if inferring the root is the goal of your study, or if the root position is controversial, you might use the reconciliation analysis itself to infer the root (see below).
Gene tree–species tree reconciliation.
  Set up the AleRax gene families file, which specifies where the gene tree samples for each gene family are to be found (see AleRax wiki at https://github.com/BenoitMorel/AleRax/wiki/Running-AleRax#families-file).
  Run an AleRax analysis using the default model and a rooted fixed species tree, using 10 cores on a machine with MPI installed:

mpiexec -np 10 alerax -f families.txt -s rooted_species_tree.newick —species-tree-search SKIP.

***Top tips***

– For analyses of protein datasets on deep evolutionary timescales, we have found that deleting (or “trimming”) poorly aligned positions (e.g. using a tool such as BMGE or trimAl) can improve phylogenetic inference.
– When selecting marker genes for species tree inference, it is important to choose genes that are single copy in most of the species analysed. In the case of duplicate genes, mixing orthologues and paralogues will confound the phylogenetic signal.
– Two useful optional AleRax commands include –trim-ratio X and –memory-savings. Not to be confused with “trimming” alignment sites, –trim-ratio X will discard some proportion X of the families with the largest number of clades in their sample of input trees; this can greatly reduce total runtime. For example, –trim-ratio 0.05 will discard the 5% of families with the largest number of clades in the input tree sample. –memory-savings will substantially reduce RAM usage at the cost of 10-20% additional runtime, which can be useful when analysing large datasets or when using machines with limited RAM.
– Remove highly similar or identical sequences in gene family alignments.
– Explore using branch-wise DTL models if the gene evolutionary process is highly heterogeneous (cf. https://github.com/BenoitMorel/AleRax/wiki/Running-AleRax#model-parametrization).
– Although the standard optimizer (GRADIENT) is fast compared to other options, it might struggle occasionally to converge to a global optimum. If your analysis allows for more runtime, we recommend using the LBFGSB optimizer via the –rec-opt flag.

### Probabilistic reconciliation-based approaches to studying microbial evolution

Probabilistic phylogenetic reconciliation can be considered a natural extension of traditional phylogenetic inference using substitution models. Just as the parameters of the substitution model include the gene tree and the relative rates of change between different nucleotide or amino acid states, the parameters of the reconciliation model describe the rooted species tree and the rates of DTL events. Reconciliation scenarios are then equivalent to substitution histories, with each sampled history representing a specific series of evolutionary events giving rise to the observed gene tree (or sequence alignment). In both cases, we sum over all possible scenarios when calculating the likelihood. As in the case of substitution models, we can choose among different reconciliation models that describe the evolutionary process in a more simple or complex way. For example, the simplest model in the phylogenetic reconciliation package AleRax [4] assumes a single set of DTL rate parameters for all genes and all branches of the species tree (Table 1). Alternatively, we might use a model in which rates vary among different clades or branches of the tree, potentially capturing important biological signal at the expense of additional model complexity and risk of overparameterization (i.e. the inclusion of additional unnecessary parameters that might increase computational cost and decrease accuracy).

The input to phylogenetic reconciliation packages varies (Table 1). Some methods (e.g. Phyldog [58], GeneRax [59]) take gene family sequence alignments as input and infer the rooted species tree as well as the reconciliations by jointly optimizing the likelihood of the reconciliation and substitution models. Most current methods use a two-step approach. First, gene trees are inferred using a standard phylogenetic reconstruction tool (perhaps IQ-TREE [40], RAxML-NG [39], or PhyloBayes [41]), which are then provided as input to the reconciliation software. Due to this inherent uncertainty of gene trees, some packages [4, 10] represent each gene family via a sample of plausible trees, obtained using bootstrapping or, in Bayesian analyses, by Markov Chain Monte Carlo sampling. Taking a sample of trees for each gene family captures this phylogenetic uncertainty, leading to reconciliations that better represent the potentially weak signal in the original sequence alignments [4]. The number of gene families reconciled in an analysis depends on the scientific question and can range from a single gene family to all available gene families on the set of genomes being analysed (see examples below).

### How phylogenetic reconciliation has been applied in microbial ecology and evolution

**Figure 2:**
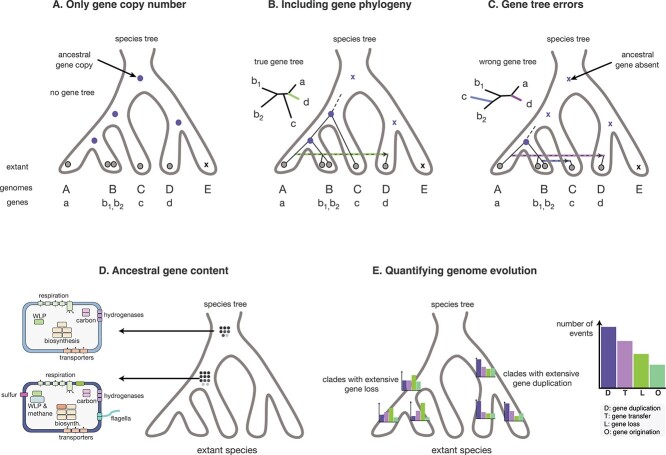
Reconstructing ancestral gene content using phylogenetic reconciliation. By “drawing” the gene tree into the species tree, phylogenetic reconciliation provides an explicit estimate of gene presence at internal nodes of the species tree and therefore ancestral gene complements. One advantage of reconciliation-based approaches to ancestral gene content inference is that the method accounts for gene transfer, as illustrated here. Grey dots denote observed gene copies in extant species, blue dots denote inferred ancestral presence of a gene, blue Xs denote inferred ancestral absence of a gene. (A) Consider a gene family broadly distributed in extant taxa. On the basis of this phylogenetic distribution alone, it appears likely that the gene traces to the root of the species tree. (B) However, a comparison of the gene tree to the species tree suggests a recent horizontal acquisition of the gene on the right hand side of the species tree root; as a result, the gene is inferred to have originated more recently. Comparison of (A) and (B) illustrates why methods based on gene copy number distribution alone may overestimate ancestral gene contents. (C) However, incorporating phylogenetic information comes at a potential cost: Errors in the reconstructed gene tree may be interpreted as additional gene transfers, so that the evolutionary age of the gene is underestimated. This case illustrates why reconciliation-based methods may tend to underestimate ancestral gene repertoires. (D) Performing reconciliation analyses for all gene families in a dataset results in the inference of gene contents at ancestral nodes, with per-family presence probabilities based upon the reconciliation model. By taking all gene families above a given probability threshold and cross-referencing with information about gene functions (e.g. based upon the COG or KEGG databases), ancestral metabolic capabilities can be inferred. (E) The reconstructed history of gene origination, DTL events on each branch of the species tree can be used to quantify genome dynamics through time, identifying periods of genome remodelling and evolutionary innovation. This figure is based on that of [120] with some modifications.

**Figure 3:**
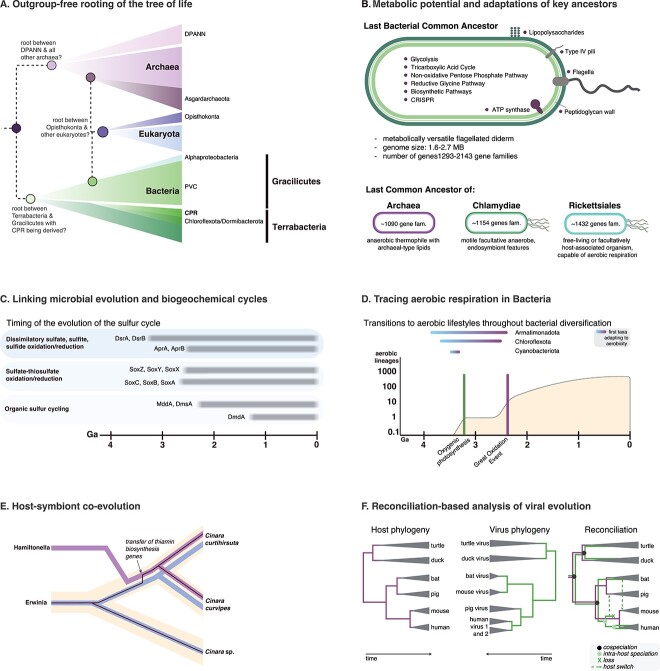
Inferences of microbial evolution from reconciliation analyses. (A) Phylogenetic reconciliation can be used to root trees without the use of an outgroup, which is useful when the outgroup is distant—as is the case when rooting entire domains of life—or when no outgroup is available, as is the case for the universal tree. The schematic tree shown in A is based on a recent timetree, [121] whereas ancestral gene sets were inferred in separate studies [56, 64] and are based on distinct approaches. (B) Reconciliation-based ancestral genome reconstruction has been used to infer the gene repertoires of key ancestors across the tree of life, including the archaea, bacteria, Chlamydiae [83] and Rickettsiales [82]. (C) Reconciling gene trees with a dated species tree enabled the earliest steps in the sulphur cycle to be discerned [89]. (D) Combining reconciliation analysis with machine learning classification of oxygen adaptation [95] enabled transitions in oxygen use to be mapped across the bacterial phylogeny; the earliest transitions were inferred to have occurred among the Cyanobacteriota, Chloroflexota, and Armatimonadota. (E) Three-level reconciliation models capturing host, endosymbiont, and gene trees can identify nested cases of host–symbiont coevolution, such as transfer of niche-relevant genes between endosymbionts of Cinara aphids [3, 104]. (F) Reconciliation of host and viral trees provided evidence for frequent host switching and within-host viral speciation in herpesviruses [122]; this panel is inspired by and adapted from Fig. 1 of the original study [122]. Although probabilistic reconciliation methods are powerful, results depend on the assumptions of the model used, the quality of the genomic information available and the taxon sampling, and it is likely that the view of early microbial evolution depicted here will be revised and updated as new, better-fitting models are developed and applied to these enduring questions.

The use of phylogenetic reconciliation methods in microbial ecology and evolution has recently gained popularity. Reconciliation-based approaches to ancestral gene content inference are particularly useful for studying microbial evolution because the reconstructions naturally incorporate phylogenetic evidence for HGT (Fig. 2). Probabilistic reconciliation methods have the additional benefit that they can accommodate phylogenetic uncertainty by averaging over possible reconciliation histories when inferring ancestral gene repertoires. In what follows, we briefly summarize some interesting applications that demonstrate how these methods can be used to link the tree of life and Earth history, reconstruct metabolic repertoires, infer past ecological transitions, and reconstruct the evolution of biogeochemical cycles (Fig. 3A–D). To date, most probabilistic reconciliation studies in microbial evolution have used ALE [10], which was the first efficient implementation of an algorithm that can account for gene tree uncertainty and model HGT. ALE has now been superseded by AleRax [4], which provides a faster, parallelizable and more flexible implementation of the original model, alongside other useful new features. In the examples below, we note when reconciliation packages other than ALE were used.

### The origin of eukaryotes and the tree of life

Phylogenetic reconciliation has recently been used to reconstruct ancestral gene repertoires among the Asgard archaea, the archaeal lineage most closely related to eukaryotes [60]. The analysis indicated that rates of gene duplication were higher in two Asgard lineages, the Lokiarchaeales and Hodarchaeales, the latter of which appear to be the closest living archaeal relatives of the eukaryotic cell. This result suggests that some of the genome dynamics that distinguish prokaryotes and eukaryotes (such as a higher rate of gene duplication in eukaryotes) might have predated the prokaryote-to-eukaryote transition [60], also recently suggested by other analyses [61]. The analysis also suggested that the archaeal lineage ancestral to eukaryotes likely lost the capacity for autotrophic growth using genes of the Wood-Ljungdahl pathway prior to entering symbiosis with the bacterial ancestor of the mitochondrion, arguing against eukaryogenesis scenarios in which the archaeal partner was a hydrogen-dependent autotroph [60].

Looking still further back in evolutionary history, phylogenetic reconciliation was used to root the bacterial phylogeny (Fig. 3A and B) [56]. The advantage of this approach is that the bacterial tree could be rooted without relying on a distant archaeal outgroup that may introduce long-branch attraction artefacts and which some have argued may not even be an outgroup to bacteria [62, 63]. Similar considerations have motivated the use of reconciliation methods to infer the root of the archaeal [64] and eukaryotic [65] domains (Fig. 3A). The root position inferred [56] provided support for a deep divide between the Terrabacteria [66, 67] and Gracilicutes, at odds with recent inferences that the Patescibacteria/CPR are basal in the bacterial domain [68, 69]. Instead, this group of ultrasmall, reduced bacteria was recovered as sister to the Chloroflexota within the Terrabacteria, in agreement with other recent inferences [70–73]. The analysis also suggested a loss of 0.47–0.56 Mb during reductive evolution along the Patescibacteria/CPR stem lineage. Interestingly, another recent reconciliation analysis [74] indicated that reductive evolution in Patescibacteria/CPR also continued in parallel more recently in their evolution.

The reconciliation analysis used to root Bacteria [56] indicated that roughly two-thirds of inferred gene transmissions were vertical (from ancestor to descendant), whereas one-third were horizontal on the species tree, identifying both a significant vertical and horizontal component of deep bacterial evolution [19]. Based on the reconstructed ancestral gene repertoire, the last bacterial common ancestor was rod-shaped, flagellated, motile, and diderm; i.e. it possessed a double membrane, suggesting that the monoderm (single-membrane) phenotypes of bacteria such as the Chloroflexota, Patescibacteria, Actinomycetota, and most Bacillota are the result of multiple independent losses of the outer membrane during terrabacterial evolution [71, 75].

### Genome evolution associated with ecological adaptation

Reconciliation analyses have also been used to study changes in gene content during historical periods of ecological adaptation, such as changes in niche or transitions from free-living to host-associated lifestyles. One of the most striking ecological transitions in evolutionary history was the evolution of the Haloarchaea—aerobic halophiles—from anaerobic methanogenic ancestors, and several reconciliation studies have investigated the genomic changes associated with this transition [76, 77]. These analyses showed that adaptation to halophilic conditions may have occurred at least four times in different archaeal lineages, with some niche-relevant genes shared among these groups by HGT, and that a reverse transition—from salt-tolerant to more moderate conditions—may have occurred in the Hikarchaeia, close relatives of the Haloarchaea [77].

Reconciliation-based reconstruction of ancestral gene repertoires was performed in Woesearchaeota [78], a lineage within the DPANN Archaea with larger genomes and greater metabolic versatility than their sister clade, the Pacearchaeota. The authors reported that the common ancestor of the two groups also had a small genome, with the acquisition of new genes, primarily via HGT, driving an increase in metabolic versatility, and transitions between host-associated and potentially free-living lifestyles in Woesearchaeota [78]. This study demonstrates the utility of tracking genome expansion and contraction across the tree of life using reconciliation methods.

Phylogenetic reconciliation has been used [79] to trace the genomic basis of niche partitioning between non-ammonia oxidising Thaumarchaeota living in acidic topsoils and subsoils in Scotland. The divergence between topsoil and subsoil-dwelling lineages corresponded to a deep phylogenetic split. A progressive expansion of gene content was observed throughout the evolution of the topsoil lineage, resulting in the extant microbes possessing significantly larger genomes than the subsoil lineage. This work also identified the horizontal acquisition of peptidases, carbohydrate-active enzymes, and an acid-tolerant ATP synthase as factors associated with ecological adaptation to topsoil.

In a different study, the same authors [80] used this approach to infer the acquisition of metabolic traits across the ecological history of Thermoplasmatota (previously the superclass Diaforarchaea [81]), an archaeal phylum that has expanded into a myriad of diverse ecosystems, including hot spring, marine, soil, sediment, and rumen environments. The reconciliation analysis revealed that essential metabolisms such as aerobic respiration and adaptation to acidic environments were likely acquired independently multiple times in the phylum’s history associated with the colonisation of new ecological niches [80].

Reconciliation methods have also been used to study the evolution of eukaryotic host association in the Chlamydiae and Rickettsiales [82, 83]. Reconstruction of the early evolution of Chlamydiae [83] suggested that the common ancestor was a facultative anaerobe that already had many of the genes needed to infect eukaryotic hosts (Fig. 3B).

The last common ancestor of Rickettsiales was predicted to be free-living or facultatively host-associated, rather than the obligate host association usually associated with members of this order. Evolution towards host association was shown to involve a general reduction in central metabolic capacity. Notably, the transition to host association corresponded with the loss of genes involved in biofilm formation and exogenous sulphate and ammonium uptake, and the gain of ATP/ADP translocase—a hallmark enzyme of energy parasitism. Furthermore, evolution towards intracellularity corresponded with a loss of amino acid biosynthesis. Conversely, evolution towards ectosymbiosis corresponded with the gain of adhesin production and export genes [82].

In addition to analyses of individual lineages, a number of reconciliation studies have focused on identifying large collections of HGT events in order to identify underlying genetic and ecological structure [84–86]. From a dataset of 960 000 trees, the parsimony method RANGER-DTL 2 was used to generate a dataset of ~2.4 million recent transfer events [86]. From these data, they observed widespread HGT with transfers inferred in 66% of gene trees. From the perspective of pangenomes [87], “accessory” genes (genes encoded by only some members of a species) were more frequently transferred than “core” genes (genes found in all members of a species). Rates of transfer also differed markedly by gene functional category and lineage, with highly abundant and co-occurring lineages exchanging the most genes [86]. These insights might inform the development of increasingly realistic reconciliation models.

### Linking microbial evolution with Earth history

Present-day biogeochemical cycles are to a large extent driven by microbial metabolism, and one emerging use of phylogenetic reconciliation is to reconstruct their historical assembly by determining the origins of the associated genes and metabolic pathways. One study [88] reconciled gene trees for nitrogen-metabolising enzymes with a dated species tree using the parsimony reconciliation method AnGST [51], reconstructing the evolution of the nitrogen cycle by dating the origin of each step. Their results suggested that nitrogen fixation mediated by molybdenum-dependent nitrogenases evolved early in life’s history (in the Archaean period, prior to 2.7Ga), whereas enzymes for denitrification evolved later, and proliferated widely by HGT after the Great Oxidation Event [88]. A similar analysis of key enzymes of sulphur metabolism using AnGST and another parsimony method, ecceTERA [52], indicated that energy conversion via sulphite reduction (or sulphide oxidation) was likely the earliest step in the evolution of the sulphur cycle [89]. ecceTERA [52] has also been used to map genes for phosphorus uptake and metabolism onto the tree of life, in order to reconstruct the bioavailability of phosphorus compounds through geological time [90]. The results suggested that phosphate uptake using a phosphate/sodium symporter was the earliest means of phosphorus acquisition among extant prokaryotes, consistent with the hypothesis that concentrations of environmental phosphate were relatively high in the Archaean period [91].

Reconciliation analysis was recently used to confirm that 2-methylhopane is a reliable biomarker for early cyanobacterial evolution, which had been contested based upon the presence of the biosynthetic gene in some Alphaproteobacteria [92]. A reconciliation analysis using the parsimony algorithm Notung [53] showed that the gene was acquired relatively recently by this latter group, suggesting that 2-methylhopane remains a reliable indicator of Cyanobacteria in older rocks [93].

One of the most significant biospheric transitions in Earth history was the Great Oxidation Event, when oxygen began to accumulate in the atmosphere, and the planet transitioned from a predominantly anaerobic to an oxidised world [94]. We recently combined machine learning with phylogenetic reconciliation to reconstruct the history of oxygen metabolism in Bacteria [95]. We used machine learning to learn the relationship between gene content and aerobic metabolism in modern taxa, then used reconciliation to trace the evolution of oxygen use phenotypes through time. Our analyses suggested that aerobic respiration evolved before the Great Oxidation Event but did not become widespread until the oxidation of the atmosphere [95], providing additional time constraints for inferring the geological history of Bacteria (Fig. 3D).

More broadly, several reconciliation methods have recently been developed that use the relative age constraints implied by patterns of donor-to-recipient gene transfer to improve the accuracy of molecular clock inferences for microbes, which have traditionally been hindered by the lack of an interpretable fossil record [96, 97]. Although the application of these methods is in its infancy, they promise to substantially refine the timescale for the early evolution of life.

These examples illustrate how phylogenetic reconciliation can provide new perspectives on evolutionary questions that have proven intractable based upon the fossil or biogeochemical records alone. However, the reverse is also true, where fossil or biogeochemical information can inform reconciliation—e.g. by providing age constraints that have to be taken into consideration by the reconciliation model [98], thus reflecting the interplay between microbial evolution and Earth history.

### Host-symbiont coevolution

Although most attention has been focused on reconciliation of gene and species trees, phylogenetic reconciliation has the potential to be applied to other systems with similar hierarchical relationships [8]. Of potential interest to microbial ecologists is the use of phylogenetic reconciliation to model host-symbiont coevolution and the evolution of microbiome composition over time, where the symbionts are the “genes” and the host is the “species.” The approach could be used to determine the extent and timescale of host-symbiont coevolution and to identify host (or symbiont) switching events [99, 100]. This has already been demonstrated for obligate symbiont–host relationships such as *Wolbachia* and arthropod hosts [101]. In a more complex microbial ecosystem, Groussin et al. [102] used phylogenetic reconciliation to show how diet and host evolution shape gut bacteria over time, supporting a role for cospeciation in mammalian gut microbiome evolution and suggesting connections to human immune diseases. By contrast, recent reconciliation analyses by Maestri et al. [103] suggested that mammalian coronaviruses arose recently and have been horizontally transmitted among distantly related hosts, finding no evidence for long-term codiversification during mammalian evolution. A promising new approach for analysing cases of host-symbiont coevolution is the TALE algorithm [3], which implements a three-level reconciliation model capturing dependencies between host lineages, their symbiotic bacteria, and symbiont genes. Initial application of this method showed that it was better able to identify gene transfers of niche-relevant metabolic genes among bacterial symbionts of *Cinara* aphids than a simpler two-level (symbiont-gene) model [3, 104] (Fig. 3E).

### Viral evolution

Reconciliation methods provide a useful framework for investigating cospeciation and coevolution between viruses and their hosts, but an interesting open question is whether, and to what extent, the conceptual model of microbial evolution implicit in reconciliation analyses (Fig. 1A) is applicable to the evolution of viruses more generally. Is it appropriate to think of viral lineages that retain coherence through time despite gene transfer or is viral evolution sufficiently dynamic that the imposition of an overarching lineage tree hinders, rather than aids, conceptual understanding, and practical phylogenetic inference? Recent advances in viral taxonomy—and the recognition of several major lineages of viruses, each united by shared core genes [105, 106]—may provide a framework with which to test these questions empirically. Recent work suggests that a lineage tree may provide a useful structure for the study of at least some lineages, including the large DNA viruses (Nucleocytoviricota, [107]). A practical challenge to reconciliation-based analyses of viral evolution is the difficulty of inferring reliable trees for viral genes, which often contain long branches that can be difficult to resolve accurately and might exhibit insufficient phylogenetic signal due to relatively short genomes in conjunction with an extremely large number of sequences [108]. However, reconciliation approaches are valuable to study virus–host coevolution and to investigate viral genome dynamics over shorter evolutionary timeframes and at shallow taxonomic levels. For example, a time-resolved reconciliation-based approach was recently implemented to determine to what extent herpesviruses evolve by coevolution (Fig. 3F), revealing that other mechanisms such as intrahost speciation, virus loss, and host switches are much more prevalent than anticipated previously (Brito et al., 2021 [122]). In another study, a phylogenetic reconciliation workflow combining the parsimony methods TreeFix-DTL [11] and RANGER-DTL [2] was used to identify recombination events in rapidly evolving viruses like SARS-CoV-2 [109].

### Using machine learning to improve reconciliation analyses

Reconciliation analyses face a major scaling challenge to handle the enormous amount of genome data now available for known microbial lineages, both in the reconciliation step itself but also in the phylogenetic inference of the underlying gene trees. However, progress is rapid in this field, and the even more rapid development of machine learning methods offers new opportunities to accelerate phylogenetic and reconciliation analyses without compromising accuracy. New methods that use machine learning to rapidly select the best-fit phylogenetic model [110, 111], to optimise analysis settings [112, 113], to efficiently search tree space [114], and to inexpensively predict bootstrap support values [115] will all benefit phylogenetic inference. One reason for the efficiency of these new methods is that machine learning algorithms can predict the results of computationally expensive likelihood calculations using cheap-to-compute input features, such as parsimony (gene) trees, which exhibit high feature importance in recent studies (70%–80%; [112, 115]). An analogous approach may prove beneficial for reconciliation, where computationally cheap input features derived from parsimony reconciliations could be used to predict the results of a full probabilistic analysis.

### Conclusions and prospects for progress

Phylogenetic reconciliation methods have emerged from the evolutionary biology community and are now becoming increasingly popular in the analysis of microbial genomes. As our examples demonstrate, reconciliation methods provide a natural analytical framework for studying microbial ecology and evolution that, in our view, is currently underutilized. We expect that these approaches will become more popular and deliver new insights into microbial diversity. There are underlying similarities between the substitution models used in traditional phylogenetics and probabilistic models for phylogenetic reconciliation, and just as with standard phylogenetic analyses, reconciliation studies will benefit from the development of improved models that better capture the patterns and processes of microbial evolution.

In addition to helping to speed up pipelines, it may also be possible to use machine learning in combination with reconciliation analyses to better model key aspects of microbial ecology and evolution. A current limitation of reconciliation methods (and indeed, most phylogenetic approaches) is that they do not consider coevolutionary interactions among gene families due to the computational complexity of doing so. Existing work that models gene co-occurrence and mutual avoidance patterns in modern bacterial pangenomes [116] might be extended to model interactions through time, in order to improve inference of ancestral gene complements. Machine learning may also be of use in inferring ancestral metabolic capabilities based upon reconstructed gene complements, as we demonstrated recently [95]. Beyond the origin of aerobic respiration, other metabolic transitions—such as the origin of photosynthesis—could be studied using the same approaches, raising the possibility of reconstructing ancient ecologies and biogeochemical cycles by integrating the genomic, fossil, and isotopic records, providing a powerful new statistical framework for studying microbial ecology and evolution through deep time.

## Data Availability

Data sharing not applicable to this article as no datasets were generated or analysed during the current study.
